# The Development and Toxicological Evaluation of Novel Polyurethane Materials

**DOI:** 10.3390/toxics13060512

**Published:** 2025-06-18

**Authors:** Maolan Zhang, Xuanran Luo, Maocai Jiang, Yu Wen, Peng Wang, Peixing Chen, Da Sun

**Affiliations:** 1 Chongqing Engineering Laboratory of Nano, Micro Biological Medicine Detection Technology, Chongqing University of Science and Technology, Chongqing 401331, China; 2019016@cqust.edu.cn (M.Z.); 17802330726@163.com (X.L.); 18723690681@163.com (M.J.); wen_yu2k@163.com (Y.W.); 18155602003@163.com (P.W.); 2 National & Local Joint Engineering Research Center for Ecological Treatment Technology of Urban Water Pollution, College of Life and Environmental Science, Wenzhou University, Wenzhou 325035, China

**Keywords:** PU, soft segment composition ratio, degradation, biological toxicity

## Abstract

Polyurethane (PU) is widely employed in the biomedical field. As application scenarios become increasingly complex, it is essential to modify PU to meet diverse requirements. Additionally, the degradation of PU is closely linked to the sustainability of its function, with degradation products having a direct impact on adjacent tissues. In this study, a novel PU containing double bonds in its main chain was developed. We investigated the influence of various ratios of soft segment composition on the degradation performance of PU, maintaining a fixed ratio of soft to hard segments and utilizing specific synthesis methods. The structure and molecular weight of the PU were analyzed using FTIR, NMR, and GPC techniques. The results of physical and chemical performance tests indicated that an increase in polycaprolactone diol (PCL diol) content within the soft segment enhanced the mechanical properties, hydrophobicity, and degradation performance of the PU. A further assessment of the degradation toxicity of PU was carried out using zebrafish as a model organism. The findings indicated that the degradation solution of PU exhibited slight toxicity to zebrafish embryonic development over prolonged degradation periods. However, it also significantly enhanced the hatching of zebrafish embryos. In summary, the novel PU developed in this study demonstrates favorable biocompatibility, and the approach of introducing reaction sites or modifying the composition of its soft segments within the molecular structure offers a promising and effective strategy to address specific application requirements.

## 1. Introduction

Degradable materials play a significant role in the biomedical field, particularly in applications such as implantable or interventional medical devices designed for repairing or replacing damaged tissues [1,2], as well as serving as sustained-release drug carrier materials [3]. Polyurethane (PU) has been extensively utilized in various medical devices since its introduction in the 1950s as a biostable material for fracture repair. Initially, research on PU focused primarily on enhancing the material’s biological stability [4]. However, its use expanded to encompass a wide range of medical devices—including guided bone repair membranes, heart valves, tissue adhesives, implantable stents, and medical catheters [5,6,7,8,9,10]. The immune response elicited by PU has garnered increasing interest from researchers, prompting efforts to modify this material [11,12,13]. In contrast to other biodegradable polymers, such as poly(lactic acid) (PLA) [14], poly(lactic-co-glycolic acid) (PLGA) [15], and poly(ε-caprolactone) (PCL) [16], PU’s flexible and adjustable chemical structure allows it to be tailored for specific tissue requirements while maintaining excellent mechanical properties, biocompatibility, processing versatility, and the absence of toxic degradation products [17].

The synthesis method of PU allows for the incorporation of raw materials with high thermal stability, excellent mechanical properties, and desirable biocompatibility or antibacterial traits to enhance the performance of PU and expand its range of applications. For instance, researchers have developed biobased PU by integrating natural substances such as cellulose or castor oil into the molecular structure of PU, resulting in improved thermal stability and mechanical properties of the material [18,19]. Xu et al. tethered small-molecule derivatives of 4-arylazo-3,5- diamino-1H-pyrazole on PU biomaterial surfaces, and the modified polymers demonstrated biocompatibility and resistance to microbial infection both in vitro and in vivo [20]. Notably, the surface properties of biomaterials, including hydrophilicity and surface morphology, significantly influence the interactions of cells, proteins, and other substances with the material surface [21,22]. For instance, high hydrophilicity and good lubricity are fundamental characteristics of medical devices such as blood vessels and human catheters. Hydrophilic surfaces can promote the desorption of adsorbed proteins, thereby decreasing the likelihood of thrombosis [23,24]. Additionally, bacteria may utilize their hydrophobic chemical groups to interact with and adhere to material surfaces [25]. Consequently, developing PU materials with enhanced hydrophilicity presents a promising avenue for advancement. Currently, the primary method for improving the hydrophilicity of PU involves incorporating hydrophilic or hydrophobic groups into the material’s molecular structure, making it more suitable for a wider range of biomedical applications. In summary, PU is developing rapidly and is widely used in the biomedical field. However, current research on PU primarily concentrates on improving its biocompatibility and mechanical properties, with insufficient emphasis on its reactivity and biotoxicology. To propel the development of PU-based medical materials, it is crucial to further modify their structures and increase the number of active reaction sites, as well as conduct comprehensive investigations into their biocompatibility, taking degradation safety into account. This will help PU to meet the practical requirements for effective biomedical applications.

Designing potential active reaction sites in PU to incorporate bioactive molecules or antibacterial agents is essential for enhancing its performance. These strategies are key to addressing the limitations of PU and broadening its applications in the biomedical field. For instance, bacteria can adhere to various polymer biomaterials and the proteins that are adsorbed on their surfaces. Therefore, one effective strategy to inhibit bacterial attachment and growth on material surfaces is to modify the surface morphology or introduce active substances during the material design process to achieve antibacterial properties [26]. On the other hand, as a type of biomaterial, PU undergoes various processes such as hydrolysis, enzymatic hydrolysis, and in vivo oxidative degradation after a certain period within the body [27,28,29]. These processes ultimately lead to a decline in device performance. Additionally, the degradation process releases different types of molecules, altering the microenvironment of surrounding tissues and potentially causing local inflammation, which can result in unsatisfactory treatment outcomes. Consequently, there has been a growing interest in the controllable degradation of materials in recent years. Prior research has explored the degradation behavior of PU concerning various compositional structures and the ratios of soft to hard segments [30,31]. However, a crucial aspect often overlooked by researchers is the biosafety of the degradation process. It is essential that the material does not produce compounds that could potentially harm tissues or lead to the accumulation of acidic substances in local environments, as this could trigger sterile inflammation. While PU demonstrates superior cell compatibility compared to other materials, long-term clinical experiences have indicated that its degradation process may still yield biologically toxic compounds [32,33]. Consequently, it is crucial to analyze the degradation process of PU and evaluate the biotoxicity of its degradation products. The zebrafish serves as a valuable model organism in life science research and has been employed across various fields, including disease modeling, drug screening, and toxicology [34,35,36]. However, there is still a notable lack of research focused on using zebrafish models to assess the toxicity of polyurethane degradation products.

In this study, we synthesized a new type of degradable PU and examined its degradation process and the safety of the degradation products in vitro. For the soft segment of the PU, we selected two raw materials, polyethylene glycol 2000 (PEG 2000) and PCL diol, in varying ratios to achieve suitable hydrophilicity and potential reaction sites. As the hard segment, we chose hexamethylene diisocyanate (HDI) due to its lower cytotoxicity compared to aromatic diisocyanates. To further increase the active reaction sites in the PU, we also used cis-2-butene-1,4-diol as a chain extender. We examined the mechanical properties and degradation behavior of synthesized PU and co-cultured different degradation solutions with zebrafish at different stages to investigate the biological safety of the material.

## 2. Experimental Procedures

### 2.1. Materials

PEG 2000 was purchased from Kelong Chemical (Chengdu, China). The following ingredients were purchased from Aladdin Biochemical Technology (Shanghai, China) and employed as received: ε-caprolactone, cis-2-butene-1,4-diol, HDI, carboxymethyl chitosan, Irgacure 2959 (I2959) and Sn(Oct)_2_. Dichloromethane, anhydrous ethanol, n-hexane, toluene, glycidyl methacrylate, and trichloromethane were all purchased from Chuandong Chemical (Chongqing, China). Among them, Toluene was distilled over freshly powdered calcium hydride under reduced pressure. *S. aureus (ATCC 6538)* bacteria were supplied by the Beijing Centers for Disease Prevention and Control (Beijing, China).

### 2.2. Preparation of Novel PU Samples

PU was produced through the two-step method that we had previously reported in [37]. Firstly, PCL diols with active reaction sites were prepared by using ε-caprolactone as the raw material and cis-2-butene-1,4-diol as the initiator and conducting the ring-opening polymerization reaction at 140 °C for a duration of 5 h under the catalysis of Sn(Oct)_2_. The obtained polymer was then purified by anhydrous ethanol and n-hexane at room temperature and dried until it reached constant weight. Then, a certain proportion of PCL diol, PEG 2000, HDI, Sn (Oct)_2_, and toluene were mixed together in a round-bottomed flask equipped with magnetic stirring. The mixture was then reacted under vacuum at a temperature of 75 °C for a duration of 4 h. After that, chain extender cis-2-butene-1,4-diol was added in an amount equal to the difference between the molar amount of HDI and the soft segment. The reaction continued at a temperature of 65 °C for 3 h. Finally, the resulting product was purified using a mixture of ethanol and n-hexane and then vacuum-dried until a constant weight was achieved at room temperature.

### 2.3. Structural Characterization

The molecular structure of PCL diol and PU was analyzed using Fourier transform infrared (FTIR) spectroscopy and nuclear magnetic resonance (^1^H NMR). Specifically, FTIR analyses of PCL diol and PU were conducted on a Perkin Elmer Spectrum GX model (Waltham, MA, USA) using KBr pellets, with measurements taken over a wavelength range of 400 to 4000 cm^−1^. Additionally, ^1^H NMR measurements were performed using a Bruker AV-4500 nuclear magnetic resonance spectrometer (Billerica, MA, USA), with Chloroform-d (CDCl_3_) as the solvent. To further assess the molecular weight of PCL diol and PU, Gel permeation chromatography with multi-angle laser light scattering (GPC, Shimadzu, Tokyo, Japan) was employed, using Tetrahydrofuran as the flow solvent.

### 2.4. Mechanical Property Test

According to the ASTMD-638 standard [38], dumbbell-shaped mechanical tensile test samples were prepared using the thermoplastic method. Each sample had an effective size of 10 × 2 × 1 mm. The tests were conducted using the CMT-1202 electronic universal testing machine (Zhuhai Sansi Taijie Electrical Equipment, Zhuhai, China) at ambient temperature and 60% relative humidity (RH), with a tensile speed of 5 mm/min. This methodology allowed us to determine the tensile strength and elongation of the PU we had synthesized. To reduce experimental errors, four sets of parallel samples were prepared for each group, and testing and analysis were conducted under consistent conditions.

### 2.5. Thermal Stability Test

Thermogravimetric analysis (TGA) was conducted using a STA449C thermal analysis system (NETZSCH, Selby, Bavaria, Germany) under a nitrogen atmosphere. The analysis was performed in a temperature range of ambient temperature to 550 °C with a heating rate of 10 °C/min.

### 2.6. Exploration of PU Side-Chain Functionalization

#### 2.6.1. Preparation of Carboxymethyl Chitosan Modified PU (CS-PU)

Carbon–carbon double bonds are highly reactive and susceptible to addition reactions. Introducing cis-2-butene-1,4-diol into the molecular structure of PU creates opportunities for the functionalization of PU side chains. The specific synthesis pathway can be found in reference [39]. A mixture of PU, glycidyl methacrylate modified chitosan (M-CMCS, synthesized according to the method described in reference of [40]), and I2959 was prepared and exposed to 365 nm UV light for 20 min to yield CS-PU.

#### 2.6.2. The Antibacterial Activity of CS-PU

The inhibition zone method was employed to evaluate the antibacterial activity of CS-PU. Specifically, certain concentrations of PU, M-CMCS, and PU-CS solution were dropped onto the drug sensitivity test papers, and the solvent was left to evaporate. Subsequently, the papers were placed on a clean workbench for a minimum of 30 min to undergo UV exposure. Next, 200 µL of *S. aureus* bacterial solution with a concentration of 10^6^ CFU/mL was taken and evenly distributed across a solid culture medium. The drug sensitivity test papers were placed in the center of the medium and incubation was performed at 37 °C for 16 h.

### 2.7. Degradation Test

#### 2.7.1. Mass Loss and Scanning Electron Microscopy (SEM) Measurement

PU films were prepared according to the solution casting method by dissolving a series of PU materials in trichloromethane at a concentration of 60 mg/mL and then pouring the solution into a circular film using a PTFE mold. The solvent was first allowed to evaporate over 48 h at room temperature and then dried in a vacuum at 30 °C until a constant weight was achieved. Subsequently, a certain volume of phosphate-buffered saline (PBS) solution and a series of PU films were added to each reagent bottle, and the mixture was incubated at a constant temperature of 37 °C and 100 rpm/min. Weekly, four vials of each PU group were collected, rinsed with distilled water, and dried in a vacuum oven to constant weight. For the other experimental groups, the PBS solution was replaced, and the degradation process continued. The weight loss ratio was calculated as follows:

(1) $$Weight\;loss\left(\%\right)=\frac{W_{0}-W_{t}}{W_{0}}\times 100\%$$

Here, W_o_ and W_t_ are the weights of the sample before and after degradation, respectively.

To investigate the changes in surface morphology of the dried films, SEM (JSM-IT2002400301, JEOL, Tokyo, Japan) was utilized.

#### 2.7.2. Determination of Contact Angle (CA)

The hydrophilicity and hydrophobicity analyses were conducted by assessing the water absorption capacity and water contact angle of PU films. To conduct these analyses, specific weights of PU films were placed in reagent bottles containing the designated volume of PBS, and the mixtures were incubated at 37 °C for 50 h. Samples were collected at 10 h intervals, rinsed with distilled water, and then weighed. The weight of the samples after absorbing distilled water was recorded as W_w_. Subsequently, the samples were vacuum-dried until reaching a constant weight, which was noted as W_t_. The water absorption rate of the PU films was calculated using the appropriate formula.

(2) $$Water\;absorption\left(\%\right)=\frac{W_{w}-W_{t}}{W_{t}}\times 100\%$$

The changes in the contact angle of water droplets on PU samples were measured using a dynamic contact angle meter (SDC-100, Dongguan Shengding, Dongguan, China). For sample preparation, PU was applied to a glass disc at a concentration of 40 mg/mL and allowed to evaporate naturally. Subsequently, the contact angle of water droplets was assessed at five different positions on the sample surface.

#### 2.7.3. pH Measurement

To detect the pH value of the medium soaked with a series of PU materials, a certain volume of distilled water was added to each reagent bottle. Then, the bottle was sealed, and the mixture was incubated at 37 °C for 8 weeks without changing the medium. The pH value of the medium was measured once a week using a PHSJ-5T (Leici, Shanghai, China). Four sets of parallel samples were prepared for each PU material, and the reported pH was the average of four samples.

### 2.8. Toxicity Experiment of Zebrafish

#### 2.8.1. Zebrafish Husbandry and Embryo Treatment

The AB-strain (wild-type) zebrafish were obtained from the China Zebrafish Resource Center (CZRC, Wuhan, China). They were cultured at approximately 28.5 °C under a 14:10 h light–dark photoperiod. Fertilized eggs were collected and washed with an embryo culture medium for subsequent biosafety evaluation of the PU degradation solution. Normal embryos, at 2 h post-fertilization, were randomly distributed into 6-well plates, with 15 embryos per well and 5 mL of PU degradation solution with different degradation times. Four parallel samples were set up in each experimental group. PBS was considered the blank control group and 50% of the treatment solution was changed daily. The embryos were continuously exposed until 72 h post-fertilization, and the toxicity endpoint of zebrafish embryos was observed and recorded every 24 h.

#### 2.8.2. Embryo Toxicity Assessment

Embryos were continuously exposed to a series of PU degradation solutions. Their development was monitored using a stereomicroscope (Stemi 2000, Carl Zeiss, Oberkochen, Germany) at 24, 48, and 72 h post-fertilization (hpf). The number of dead and hatched individuals was recorded to calculate the survival and hatching rates of zebrafish. Indicators for embryonic death were determined based on the following criteria: (1) low transparency and refractive index, (2) absence of heartbeat, and (3) inadequate formation of body segments [41].

## 3. Results and Discussion

### 3.1. Structural Characteristics of PCL Diol and PU

**Figure 1:**
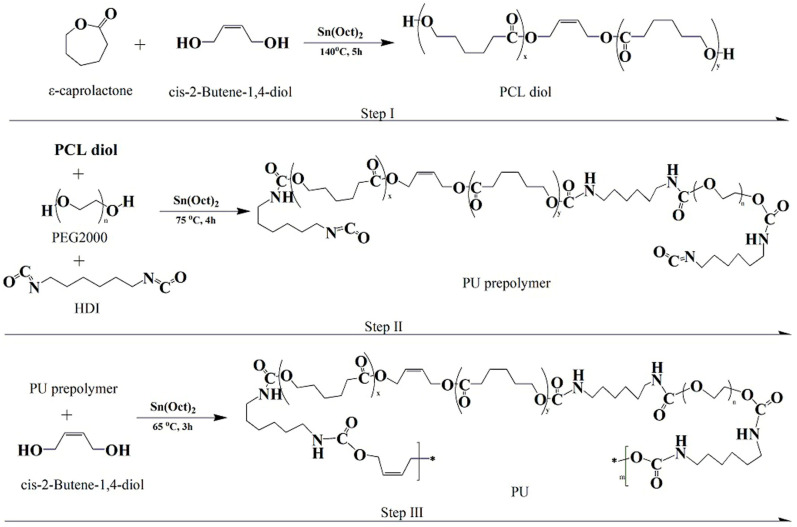
Synthesis of PU from PCL diol, PEG 2000, and cis-2-butene-1,4-diol.

We employed a traditional two-step method for the synthesis of PU, as depicted in Figure 1, which illustrates both the synthesis process and the chemical structure of PU. In the synthesis of PCL diol, the molar ratio of ε-caprolactone to cis-2-butene-1,4-diol was set at 25:1. For the soft segment, the ratios of PCL diol to PEG 2000 were 4:6, 1 and 6:4, respectively. The hard segment was composed of HDI and cis-2-butene-1,4-diol, with the overall ratio of the soft segment, HDI, and cis-2-butene-1,4-diol being 1:1.2:0.2. These values were selected based on our prior research findings [37].

**Figure 2:**
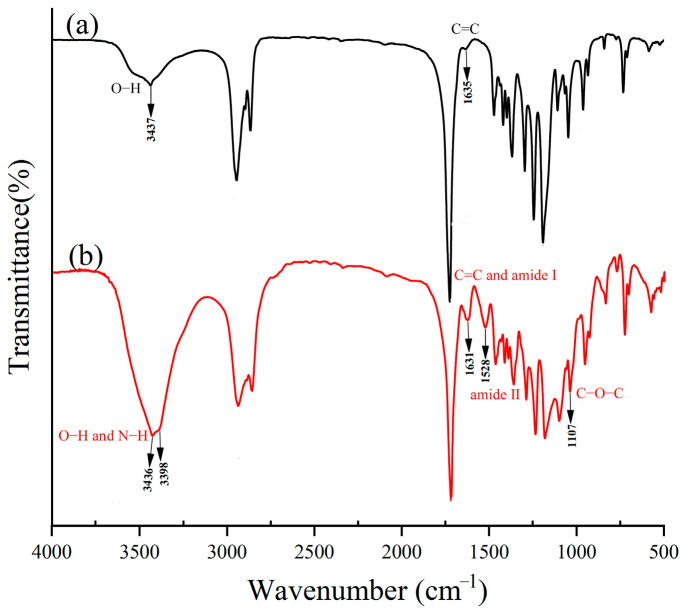
FTIR spectra of PCL diol (**a**) and PU (**b**).

Figure 2 displays the FTIR spectra of PCL diol and PU. The stretching vibration peaks at 3437 cm^−1^, 1724 cm^−1^, and 1635 cm^−1^ corresponded to the O-H, C=O, and C=C stretching vibrations, respectively, as depicted in Figure 2a. Additionally, the C-H in-plane bending absorption and C=O stretching vibration absorption peaks were observed at 1471 cm^−1^ and 1191 cm^−1^, respectively. In Figure 2b, compared to PCL diol, the stretching vibration peaks of end O-H and hard segment N-H appeared at 3436 cm^−1^ and 3398 cm^−1^, respectively. The absorption peak at 1728 cm^−1^ was attributed to the amide carbonyl stretching vibration peak. The increase in peak intensity at 1631 cm^−1^ may be due to the further introduction of C=C in the hard segment, as well as the characteristic absorption peak of the amide I of urethane. The new absorption peak appearing around 1528 cm^−1^ belonged to amide II of urethane. In addition, the peak appearing around 1107 cm^−1^ was attributed to the -C-O-C characteristic absorption peak in PEG 2000. All these results indicate that PU was successfully prepared.

**Figure 3:**
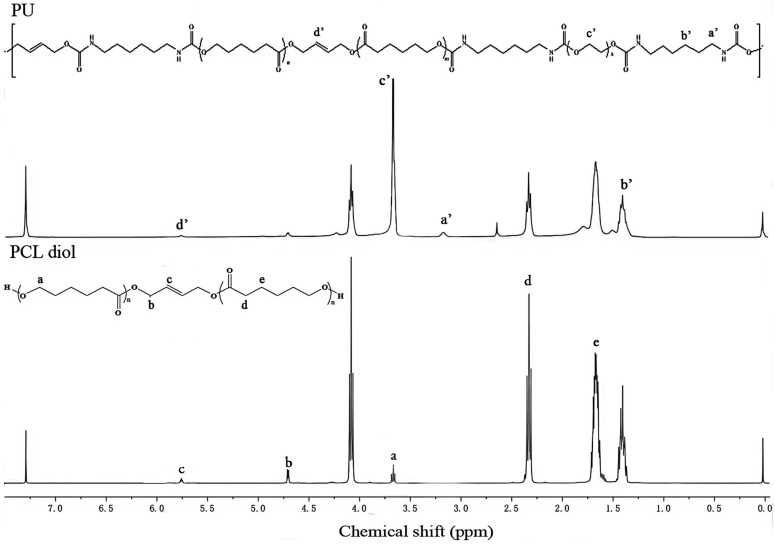
^1^H NMR spectra of PCL diol and PU.

The ^1^H NMR spectra of PCL diol and PU are displayed in Figure 3. The characteristic peaks of -O-CH_2_- and -CH_2_-CO- in PCL diol are visible at 4.72 ppm and 2.27 ppm, respectively. The hydrogen element on the double bond in cis-2-butene-1,4-diol has a characteristic chemical shift of 5.78 ppm, indicating the successful preparation of PCL diol. The peak of the methylene group in HDI appeared at 3.32 ppm and 1.13 ppm. The absorption peak around 3.69 ppm was characteristic of a chemical shift of -O-CH_2_-CH_2_-O- in PEG 2000. Additionally, the small peak of around 7.6 ppm was attributed to the chemical shift of the active hydrogen of the amide bond. The absorption peak of active hydrogen on the terminal hydroxyl group was around 2.5 ppm, confirming the successful preparation of PU. Moreover, the molecular weight of PCL diol is 6250, whereas the Mn of PU series is approximately 4.8 × 10^4^. This further confirms the success of the polymerization reaction.

### 3.2. Mechanical Property Analysis

**Figure 4:**
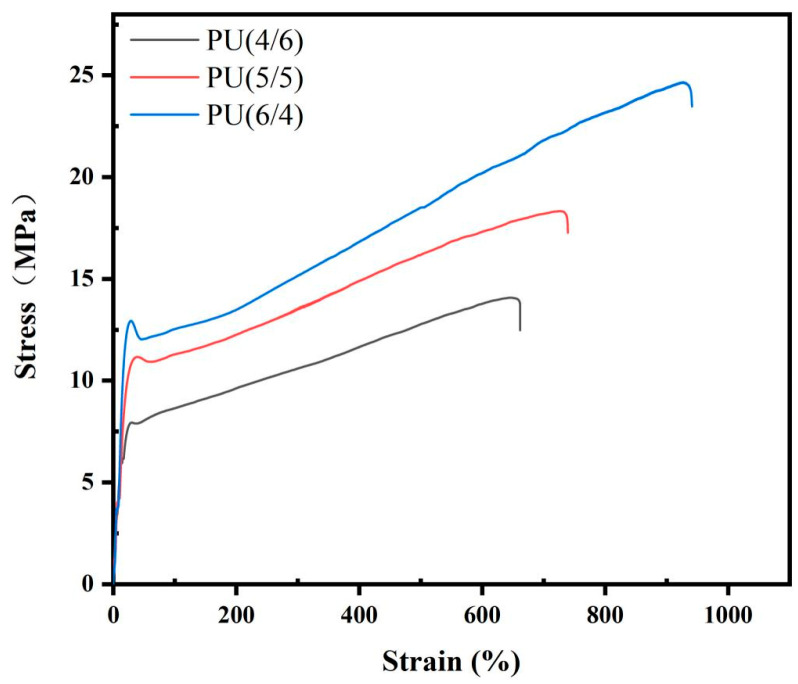
Tensile properties of a series of PU materials.

The hard-phase region of PU comprises HDI and cis-2-butene-1,4-diol, while the soft-phase region contains varying proportions of PCL diol and PEG 2000. The hard segments of PU act as physical crosslinking points through hydrogen bonding, which contributes to its superior mechanical properties. Conversely, the soft phase of PU enhances its fracture elongation and elasticity [42,43]. The mechanical properties of PU, as determined by a universal testing machine, are illustrated in Figure 4 and detailed in Table 1. It is clear that the proportion of PCL diol and PEG 2000 within the soft segment significantly influenced the material’s mechanical characteristics. As the PCL diol content in the soft segment increased, both the elastic modulus and tensile strength, along with elongation at break, showed consistent improvement. This is related to the favorable mechanical properties of PCL itself. Specifically, when the ratio of PCL diol to PEG 2000 shifted from 4:6 to 6:4 while maintaining a constant ratio between soft and hard segments, the elastic modulus increased from 31.25 to 58.34 MPa, and the tensile strength increased from 12.47 to 23.46 MPa. Notably, with a 6:4 ratio of PCL diol to PEG 2000, the elongation at break reached an impressive 941%, highlighting the close relationship between PCL diol content in the soft segment and the mechanical properties of PU. Based on existing literature reports, this study concludes that by adjusting the ratio of soft and hard segments and selecting different compositions for the soft segments, it is possible to produce biomedical PUs with a range of mechanical properties tailored to meet clinical application requirements.

### 3.3. Thermogravimetric (TG) Analysis

**Figure 5:**
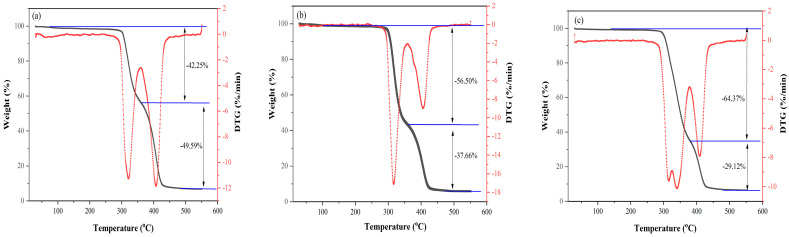
TGA and DTG curves of a series of PU materials: (**a**). PU(4/6); (**b**). PU(5/5); (**c**). PU(6/4).

The thermal decomposition of PU is a complicated process that is influenced by the type and quantity of soft and hard segments involved. Two-stage TG curves were observed for the PU materials, as shown in Figure 5. The first stage began at approximately 300 °C, while the second stage occurred between 360 °C and 430 °C. The weight loss associated with these two stages aligned with the weight fractions of the PCL diol and PEG 2000 present in the PU soft segment. As the proportion of PCL diol in the soft segment increased from 40% to 60%, the weight loss during the first stage progressively increased from 42.25% to 64.37%. In contrast, the weight loss in the second stage decreased from 49.59% to 29.12%. This indicates that the degradation of PU initiates with the breakdown of ester bonds in the PCL diol chain segment, followed by the degradation of ether bonds and methylene groups in PEG 2000. Additionally, it is noteworthy that the PU synthesized in our research institute demonstrated excellent thermal stability at ambient temperatures, which will facilitate their subsequent processing.

### 3.4. PU Side-Chain Functionalization

**Figure 6:**
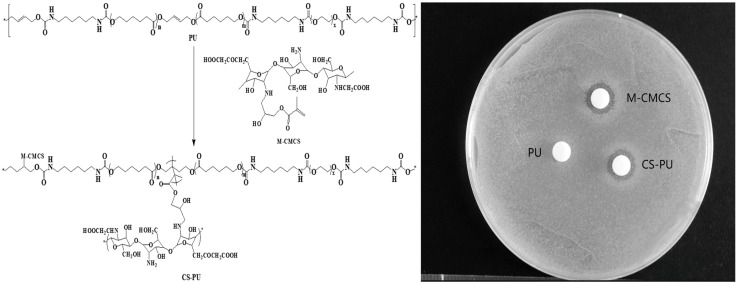
Reaction process and antibacterial performance evaluation of PU, M-CMCS, and CS-PU.

Figure 6 presents the results of the sensitivity tests conducted on *S. aureus* using PU, M-CMCS, and CS-PU, employing the inhibition zone method. The figure reveals the absence of an antibacterial zone surrounding the PU filter paper, indicating that PU itself does not possess antibacterial properties. In contrast, both M-CMCS and CS-PU exhibited distinct antibacterial zones, suggesting that M-CMCS was successfully incorporated into the side chains of PU. This finding confirms that it is possible to introduce active substances or functional groups through double bonds in PU to fulfill specific application requirements.

### 3.5. Degradation Analysis

#### 3.5.1. Mass Loss

**Figure 7:**
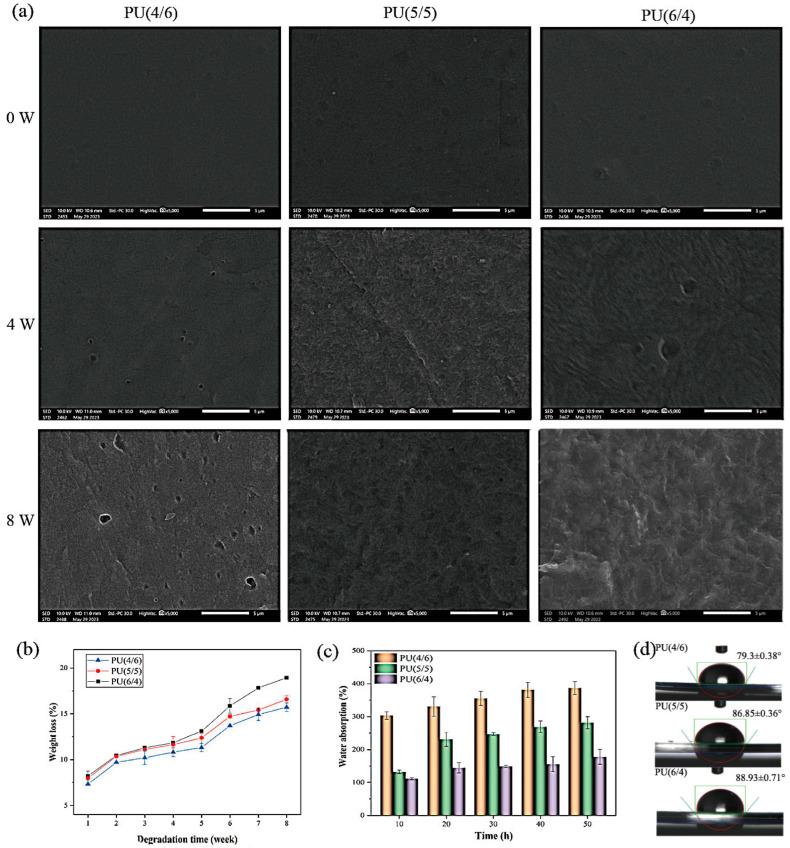
Surface morphology (**a**), weight loss rate (**b**), water absorption (**c**), and water contact angle (**d**) of PUs.

Over time, bonds within polymer chains can break, resulting in a loss of structural integrity, a decrease in weight, and the release of various small molecular substances. The presence of a degradation medium can further accelerate this process. Thus, it is imperative to assess the degradation performance of materials in vitro. Such evaluations enhance our understanding of the degradation process, offering valuable insights for studying the pattern and progression of PU degradation. Figure 7a,b depict the surface morphology and weight loss rate of the PU film material over time. Prior to degradation, the surfaces of the PU films were smooth and dense, as shown in Figure 7a. However, as the degradation duration increased, the surfaces of the films became uneven and exhibited microporous structures by the fourth week. This phenomenon became even more pronounced by the eighth week. When comparing PU (4/6), PU (5/5), and PU (6/4), it is clear that the surface roughness and micropore presence increased with higher PCL diol content in the PU soft segment. This suggests that degradation in the series of PU films primarily occurred within the PCL diol chain segments. These findings are consistent with previous studies indicating that the ester bonds in the soft segment of PU are prone to hydrolysis, leading to the formation of carboxylic acids and alcohols. The carboxylic acid generated as a decomposition product can induce self-catalytic degradation of the ester bonds, which further accelerates the material’s decomposition [44]. In Figure 7b, it is evident that the degradation of the PU can be categorized into two stages. The initial stage was the hydration stage of degradation, which progressed slowly over a period of 5 weeks. During this stage, preliminary degradation occurred, although the PU remained undissolved. In the subsequent stage, the series of PU films transitioned into the degradation and dissolution phases, marked by a rapid increase in the weight loss rate. A comparison among the series of PU films reveals that the weight loss rate increased as the proportion of PCL diol in the PU soft segment increased. This finding aligns with the results observed from SEM analysis, indicating that the degradation rate of the series of PU films was positively correlated with the PCL diol content in the soft segment of PU.

#### 3.5.2. The Analysis of Hydrophilicity and Hydrophobicity

The hydrophilic and hydrophobic properties of polymers have a significant impact on both the material characteristics and its application in the biomedical field. These properties can affect cell adhesion and protein interaction, making it essential to investigate them across various PU materials. Such studies will provide a strong foundation for the practical application of these materials and hold significant importance in the field. In Figure 7c,d, the water absorption rate and static water contact angle for the series of PU films are displayed. Specifically, among the different PU films, PU (4/6) showed the highest water absorption rate and the lowest water contact angle, indicating its strongest hydrophilicity. This characteristic is closely linked to the substantial presence of hydrophilic PEG 2000 segments within the soft segment of PU (4/6). When this PU came into contact with water, the PEG 2000 segments were able to form hydrogen bonds with water molecules, resulting in a reduced contact angle and enhanced water absorption on the membrane surface. In contrast, when the content of hydrophobic segments such as PCL diol increased, as is the case with PU (6/4), the material exhibited higher water contact angles and lower water absorption rates. Additionally, while the urethane present in the hard segment of PU does contribute to the hydrophilicity of the material, the primary differences in hydrophilicity among the various PU films in this study arise from the differing ratios of hydrophilic and hydrophobic segments in the soft segments, given that the ratio of soft to hard segments remains constant. By adjusting the proportions of hydrophilic chain segments or modifying the ratio of soft to hard segments in the PU, it is possible to create materials with varying hydrophilic and hydrophobic properties, thereby meeting the specific requirements of biomedical applications.

#### 3.5.3. pH Change

**Figure 8:**
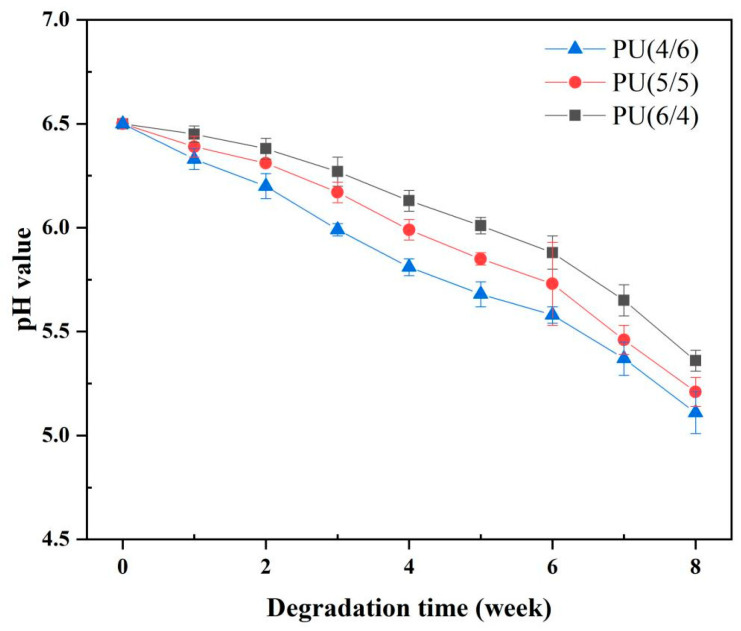
Changes in pH value with degradation time for PU.

Figure 8 illustrates the relationship between the pH level of the medium and the time required for the series of PU films to degrade during the degradation process. All experiments were conducted over a duration of 8 weeks, employing distilled water with a pH of 6.5 as the hydrolysis medium. To accurately evaluate the impact of the environmental medium and the properties of the polymer on pH changes during degradation, the medium was not altered throughout the process. Based on the graph, it can be observed that the degradation pattern of the series of PU films is consistent, and the degradation process has two stages: the hydration phase from weeks 1 to 5, followed by the degradation phase from weeks 6 to 8. In the initial 5 weeks, water molecules infiltrated the material through intermolecular forces, which led to the relaxation of polymer chains and minimal segment breakage. Consequently, the pH value of the medium exhibited only a slight decrease. However, from week 6 onward, a significant decline in pH was observed due to the extensive hydrolysis of the chemical bonds in the polymer backbone. This process not only led to the breakage of the chain segments but also generated acidic byproducts, resulting in acid-induced catalytic degradation [45]. Furthermore, when comparing the different PU films, it can be observed that the pH value decreased more sharply from the 6th week onwards as the proportion of PCL diol within the PU soft segment increased, indicating that degradation mainly occurred within the PCL diol chain segment during this stage.

### 3.6. Zebrafish Embryo Analysis

**Figure 9:**
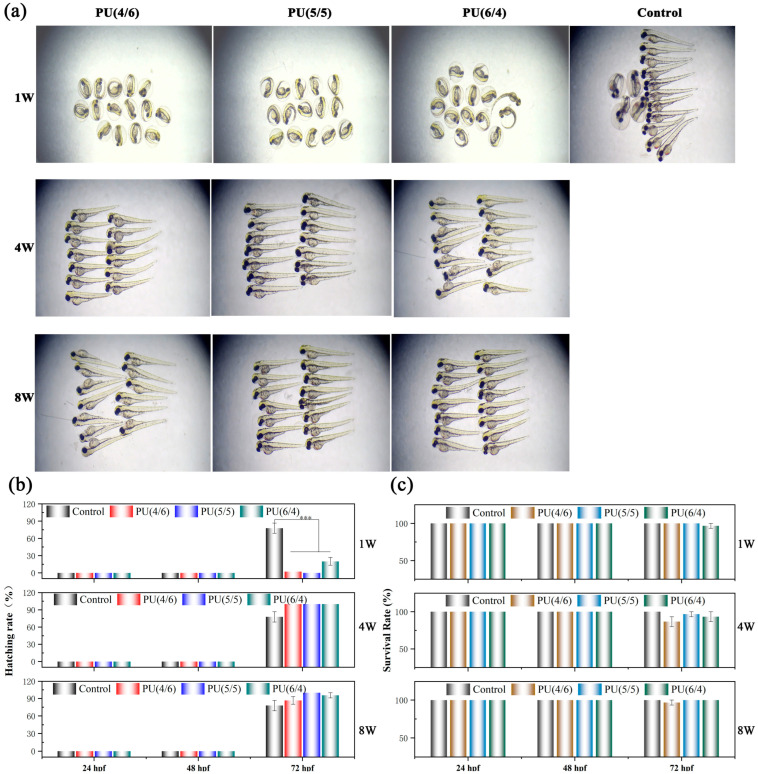
Embryo toxicity induced by PU degradation solution: (**a**) after co-incubating for 72 hpf with degradation solutions of varying degradation times; (**b**) hatching rate; (**c**) survival rate. Values represent the mean SD of four replicates (nˆ 4, 15 embryos/replicate). *** *p* < 0.001.

The zebrafish is an established model organism recognized by the International Organization for Standardization. It has several advantages, including a high level of genomic homology with humans and physiological characteristics that resemble those of mammals during development. Consequently, this study utilized zebrafish embryos to evaluate the toxicity of PU degradation solutions. The impact of a series of PU degradation solutions with various time periods on the embryonic development of zebrafish is illustrated in Figure 9. As indicated in Figure 9a, the development of zebrafish in the control group and each experimental group appeared largely normal, featuring straight spines. However, in comparison to the control group, the degradation solution derived from the series of PU materials over the course of one week resulted in a delay in embryonic development. It is noteworthy that, as the degradation time of the PU material increased, the resulting degradation solution positively impacted the hatching rate of zebrafish, particularly evident in the fourth week, as demonstrated in Figure 9b. Figure 9c illustrates the effects of the various PU degradation solutions on the survival rate of zebrafish. When compared to the control group, these PU degradation solutions caused a slight decrease in the survival rate of zebrafish, especially after four weeks of degradation. In experiments where zebrafish embryos were co-cultured for 72 hpf, a small number of embryos may have died. Interestingly, the PU degradation solution that underwent eight weeks of degradation had a minimal effect on the survival rate of zebrafish embryos. Previous analyses indicate that, after approximately four weeks of degradation, PU releases a small quantity of macromolecular alcohols or carboxylic acids, leading to an acidic degradation solution. While this acidic environment promotes hatching in zebrafish, prolonged exposure can adversely affect their survival rate, particularly when the pH of the solution drops below 6.0, as there is a significant risk of mortality at pH values below this threshold. As the degradation time of PU films continued (eight weeks), the hard segments of the PU material gradually broke down, producing alkaline substances. This interaction among the degradation products ultimately mitigated their influence on both the hatching and survival rates of zebrafish embryos. The specific mechanism of action is still being studied. Additionally, whether the degradation solution of PU will have other toxic effects on zebrafish, such as impacts on reproductive development, behavior, angiogenesis, and the central nervous system, remains to be determined. Further research is needed to explore these potential effects and the corresponding mechanisms involved.

## 4. Conclusions

PU is a versatile polymer material widely utilized across various sectors, including chemical, textile, medical, aerospace, etc. In the biomedical field, as the applications of PU expand, the demands placed on this material also increase. Specifically, an ideal biomedical PU material should possess adequate mechanical strength, hydrophilicity, safety, non-toxicity, controllable degradation performance, and active sites for potential further reactions. As a result, researchers are developing innovative types of biomedical PUs and assessing their physical, chemical, and biological properties. In this study, a novel amphiphilic PU was synthesized with a molecular backbone featuring double bonds for further reactions. By regulating the composition of the soft segment, the physical and chemical properties of PU can be regulated. Specifically, enhancing the proportion of PCL diol chain segments within the soft segment of PU can significantly improve its mechanical properties, refine thermal stability, and boost both hydrophobicity and degradation performance. After four weeks of degradation, the acidic byproducts generated by PU facilitated the hatching of zebrafish embryos while also influencing their survival rates. However, as the degradation process continued beyond this timeframe, the beneficial effects began to diminish. These results provide evidence for the expanded medical applications of PU and will aid in exploring how PU degradation products influence the growth and development of zebrafish.

## Figures and Tables

**Table 1:** Mechanical properties of a series of PU materials.

Sample	Young’s Modulus (MPa)	Tensile Strength (MPa)	Elongation at Break (%)
PU(6:4)	58.34	23.46	941
PU(5:5)	39.16	17.27	739
PU(4:6)	31.25	12.47	662

## Data Availability

The original contributions presented in this study are included in the article. Further inquiries can be directed to the corresponding authors.
